# Establishment of an In-House Indirect Enzyme-Linked Immunosorbent Assay to Detect Antibodies Against African Horse Sickness Based on Monovalent and Polyvalent Live Attenuated Vaccines During the First Outbreak in Thailand

**DOI:** 10.3390/ani15101433

**Published:** 2025-05-15

**Authors:** Darsaniya Punyadarsaniya, Machimaporn Taesuji, Khate Rattanamas, Sakchai Ruenphet

**Affiliations:** 1 Immunology and Virology Department, Mahanakorn University of Technology, Bangkok 10530, Thailand; darsaniya_p@yahoo.de; 2 Animal Biotechnology, Mahanakorn University of Technology, Bangkok 10530, Thailand; machimapornt@gmail.com (M.T.); r.khate73@gmail.com (K.R.)

**Keywords:** African horse sickness, antibody, enzyme-linked immunosorbent assay (ELISA), live attenuated vaccine, sensitivity, specificity

## Abstract

Standard serological tests post-vaccination, such as enzyme-linked immunosorbent assay (ELISA), complement fixation, and virus neutralization, are crucial for monitoring African horse sickness (AHS). However, the availability of commercial test kits such as blocking ELISA varies by regions; while they are commonly used in Africa and Europe, their limited availability and high cost in Thailand present significant challenges. Therefore, this study aimed to evaluate an alternative approach using an in-house indirect ELISA based on cell-based monovalent and polyvalent strains of live attenuated AHS virus. This method addresses the cost and accessibility issues faced in Thailand. This study demonstrated promising results: the in-house indirect ELISA showed analytical sensitivity and specificity values of 88.30% and 67.02% for monovalent strains and 87.23% and 84.04% for polyvalent strains, respectively, compared to the blocking ELISA. These findings underscore the efficacy of the in-house ELISA as a viable serodiagnostic tool for AHS. Furthermore, the polyvalent antigen-based in-house indirect ELISA proved to be a reliable alternative for AHS monitoring, particularly in vaccinated horses, offering enhanced specificity. Additionally, this method is simpler, cheaper, faster, and more convenient than blocking ELISA and serum neutralization tests, making it a practical choice for routine AHS surveillance.

## Simple Summary

Serological tests are crucial for monitoring antibodies following African horse sickness (AHS) vaccination, especially during an outbreak. However, commercial test kits, such as blocking enzyme-linked immunosorbent assay (ELISA), are not widely available in Thailand due to their high cost and limited accessibility. To address this limitation, this study assessed an alternative approach using an in-house indirect ELISA based on monovalent and polyvalent strains of live attenuated AHS vaccine. This study found that the in-house ELISA performed effectively, demonstrating high sensitivity and specificity, particularly when using the polyvalent antigen. Furthermore, this method is simpler, cheaper, faster, and more convenient than blocking ELISA and serum neutralization tests, making it ideal for routine AHS surveillance.

## 1. Introduction

African horse sickness (AHS) is a fatal vector-borne disease that affects all Equidae species including horses, mules, donkeys, and zebras [1,2]. Classified as a notifiable disease by the World Organization for Animal Health (WOAH, formerly the OIE), AHS underscores its global importance. The disease is caused by the AHS virus (AHSV), an RNA virus belonging to the *Reoviridae* family, *Orbivirus* genus [3]. AHSV is classified into nine serotypes, which are distinguished by the viral capsid protein-2 (VP-2) [4,5,6,7]. AHS is endemic in Africa and remains a significant health and trade concern for equids [8,9]. In March 2020, AHS emerged in new regions following an outbreak in northeastern Thailand, confirming the presence of AHSV serotype 1 [10,11,12,13]. The economic consequences of an AHS outbreak extend beyond increased mortality rates in horses, involving movement restrictions, culling of infected animals, vector control measures, and vaccination strategies [14,15,16].

In countries where AHS is endemic or emerging, vaccination remains the most effective and reliable method of prevention. Due to the absence of specific treatments for AHS, disease management primarily involves rest, supportive therapy, and general care. Vaccination of at-risk equids represents the most effective strategy for preventing and controlling the disease [17]. Live attenuated vaccines, which offer broad protection against all nine AHSV serotypes, are commercially available and produced by Onderstepoort Biologic Products. These vaccines are provided as two polyvalent formulations: a trivalent vaccine containing AHSV-1, AHSV-3, and AHSV-4, and a tetravalent vaccine containing AHSV-2, AHSV-6, AHSV-7, and AHSV-8 [18,19,20]. Despite their proven efficacy, live attenuated vaccines raise concerns regarding potential reversion to virulence, transmission, reassortment with field strains, and difficulties in differentiating infected from vaccinated animals [18,21,22].

To obtain OIE recognition of AHS-free status, member countries must demonstrate the absence of reported cases of AHSV infection for at least two years and must have ceased routine AHS vaccinations for the previous year [23]. Thailand, which has reported no AHS cases since September 2020 and halted vaccinations in 2021, achieved AHS-free status, a designation confirmed by the OIE in March 2023 [24]. Accurate documentation of vaccination campaigns, particularly the proportion of vaccinated animals relative to registered equids, is crucial for demonstrating effective disease control. Additionally, standard serological tests, including enzyme-linked immunosorbent assay (ELISA), complement fixation, and virus neutralization, are essential for monitoring AHS post-vaccination [23]. However, while commercial test kits such as blocking ELISA are available in Africa and Europe, their limited availability and high cost in Thailand present significant challenges [25].

This study aimed to evaluate the sensitivity and specificity of an in-house indirect ELISA for detecting anti-AHSV antibodies, utilizing monovalent and polyvalent strains of live attenuated AHSV in a cell-based system.

## 2. Materials and Methods

### 2.1. Horse Sera and Animal Ethics

A total of 94 horse sera, collected between 2018 and 2019, prior to the AHS outbreak in Thailand, and another 94 sera from horses vaccinated with a live attenuated AHS vaccine at least one month post-vaccination during the outbreak, were used for antibody detection in this study. The sera were provided by the First Livestock and Agriculture Division, Veterinary and Remount Department of the Royal Thai Army (Kanchanaburi Province), Sap Takhian Cowboy City, and KC Horse Farm (Nakhon Sawan Province). All samples were stored at −30 °C prior to testing.

The positive serum control was derived from horses that had been boosted with the AHS vaccine and subsequently confirmed positive by blocking ELISA. The negative serum control was obtained from horses prior to the AHS outbreak in Thailand and was also validated using blocking ELISA.

The experimental and animal use protocols were approved by the Animal Care and Use Committee of Mahanakorn University of Technology (ethical approval number ACUC-MUT-2021/003).

### 2.2. Commercial ELISA Test Kit

The blocking ELISA test kit (INGEZIM AHS COMPAC PLUS, INGENASA Madrid, Spain) was employed in this study for comparative analysis. It was generously provided by Perfect Companion Group Co., Ltd., Bangkok, Thailand. This assay functions by preventing the interaction between the recombinant VP7 protein, adsorbed onto the ELISA microplate, and a peroxidase-conjugated AHS-VP7-specific monoclonal antibody (mAb). VP7-specific antibodies in serum samples inhibit this interaction, thereby reducing the enzymatic reaction between peroxidase and the colorimetric enzyme substrate, resulting in a measurable color change. The optical density of the reactions was recorded, and the results were expressed as the blocking percentage, reflecting the degree to which the serum sample inhibited the conjugate–antigen reaction. All tests were conducted in accordance with the manufacturer’s instructions.

### 2.3. AHSV Antigen Preparation

Both monovalent (serotype 1) and polyvalent (serotypes 1, 3, and 4) forms of the live attenuated AHS vaccine (Onderstepoort Biological Products, Pretoria, South Africa) were used as viral seed stocks for propagation in African green monkey kidney (Vero) cells. Each vaccine strain was inoculated onto a monolayer of Vero cells to initiate viral propagation. After a 30 min incubation at 37 °C in a CO_2_ incubator, the inoculum was removed, and the maintenance medium (MM), containing Dulbecco’s Modified Eagle’s Medium (Gibco, Thermo Fisher Scientific, NY, USA) supplemented with streptomycin (100 μg/mL) and amphotericin B (0.5 μg/mL), was added for further incubation. Once the cytopathic effect (CPE) was observed, the infected cells were harvested and centrifuged at 2000× *g* for 10 min. The resulting cell pellet was retained, subjected to three cycles of freezing and thawing, and stored at −80 °C for subsequent antigen coating.

### 2.4. Indirect ELISA

Each stock virus (monovalent and polyvalent strains) was used to coat a 96-well ELISA microplate (Nunc-microwell, Thermo Scientific, Suzhou, China) in carbonate buffer (0.05 M NaHCO_3_, pH 9.6) and incubated at 4 °C overnight. To prevent non-specific binding, the coated plates were blocked with 1% skim milk in phosphate-buffered saline (PBS, pH 7.4) and incubated for 1 h on a microplate shaker. The plates were then washed three times with PBS-T (PBS containing 0.05% Tween 20).

Mock (non-infected) cells were included as background controls to assess non-specific binding and background signals. These cells were treated under the same conditions as the infected cells, but were not exposed to the AHS virus, thus providing a baseline for absorbance measurements. By subtracting the absorbance values from the mock wells, we ensured that any signal observed in the experimental wells was specifically attributed to antibody binding to the AHS virus antigen.

**Figure 1:**
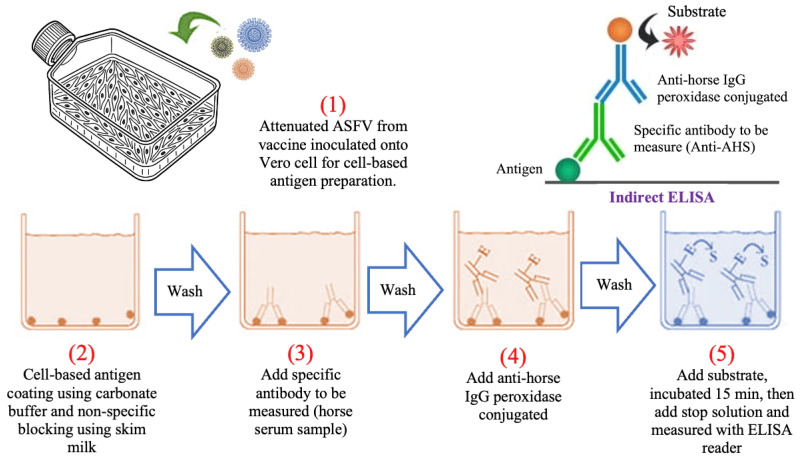
Schematic diagram illustrating a cell-based indirect enzyme-linked immunosorbent assay for the detection of African horse sickness antibodies in horse serum.

Next, diluted horse sera were added to the wells and incubated at room temperature on a shaker for 1 h. After washing three times with PBS-T to remove unbound components, a peroxidase-conjugated anti-horse IgG antibody (Rockland Immunochemicals Inc., Limerick, PA, USA) was added and incubated for 1 h at room temperature. Following further washing, the plate was treated with substrate (KPL TMB microwell peroxidase substrate system, Sera Care, Milford, MA, USA), incubated for 15 min at room temperature, and the reaction was stopped with 1 N HCl. Absorbance at 450 nm was measured using an automatic ELISA plate reader (AccuReader, Metertech, Taipei, Taiwan) and expressed as optical density (Figure 1). For each indirect ELISA plate, both AHS-positive and AHS-negative serum controls were included to calibrate the evaluation of all serum samples. The results from these controls were used to accurately calibrate the indirect ELISA.

Additionally, to assess the specificity of the in-house indirect ELISA and evaluate potential cross-reactivity, positive serum samples for equine encephalosis, equine influenza, and equine infectious anemia were tested. These serum samples were kindly provided by Dr. Nutnaree Kunanusont from the Clinic for Horse, Mahanakorn University of Technology, Thailand.

### 2.5. Standardization of the Indirect ELISA Method

A checkerboard titration was performed to determine the optimal conditions for the indirect ELISA reaction [26]. Each stock virus was applied to the ELISA plate at serial dilutions of 1:10, 1:20, 1:40, 1:80, 1:160, and 1:320 to evaluate different concentrations and identify the optimal dilution for the assay. Serum samples from negative and positive controls, confirmed by blocking ELISA, were serially diluted in PBS at concentrations of 1:50, 1:100, 1:200, 1:400, 1:800, and 1:1600 to establish a dilution series for comparing serum reactivity at various concentrations.

### 2.6. Calculation of Cut-Off Value

The optical density (OD) at a wavelength of 450 nm was measured using the indirect ELISA. The cut-off value was calculated according to the methodology described in the OIE Manual for AHSV, employing indirect ELISA [27] and was determined using the following formula: the mean absorbance of negative controls plus the standard deviation derived from a group of negative sera. Test samples with absorbance values exceeding the cut-off value plus 0.15 were considered positive, while those with lower values were classified as negative.

### 2.7. Analysis

The performance of the in-house ELISA was evaluated and expressed as percentages to assess its effectiveness. Sensitivity and specificity were determined by comparing the ELISA results obtained using monovalent and polyvalent live attenuated AHS vaccine with those from the blocking ELISA to validate the in-house assay. Results that correlated with the blocking ELISA were classified as true positives or true negatives, while those that did not correlate were classified as false negatives or false positives. The numbers of true positives, false positives, true negatives, and false negatives for both monovalent and polyvalent vaccines were recorded based on comparisons with the blocking ELISA for further analysis. Sensitivity and specificity were calculated using standard evaluation formulas to quantify the performance of the in-house ELISA:

$$Sensitivity=\frac{No.\;of\;true\;positive}{No.\;of\;true\;positive+No.\;of\;false\;negative}\times 100$$

$$Specificity=\frac{No.\;of\;true\;negative}{No.\;of\;true\;negative+No.\;of\;false\;positive}\times 100$$

### 2.8. Intra- and Inter-Assay Variability

To assess the reproducibility and reliability of the in-house indirect ELISA, both intra-assay and inter-assay variability were evaluated. Intra-assay variability was determined by performing duplicate assays on the same plate, utilizing the same batch of reagents and samples. Each sample was tested in two separate wells within the same assay, and the coefficient of variation (CV) was calculated to quantify the consistency within the assay. For inter-assay variability, the same set of samples was tested on different days using freshly prepared reagents and new ELISA plates. The consistency of results across multiple experimental runs was assessed by comparing the CV values for each assay.

## 3. Results

The performances of the in-house indirect ELISA utilizing both monovalent and polyvalent live attenuated AHS vaccines were compared with that of the blocking ELISA. Optical density (OD) values were measured at 450 nm from the checkerboard titration experiments. To ensure measurement accuracy and minimize non-specific interference, mock (non-infected) cells were used as background controls. These cells were treated under identical conditions to the infected cells, but were not exposed to the AHS virus. The absorbance readings from the mock wells provided a baseline, which accounted for non-specific binding or interference that could affect the assay. The OD values were subsequently calculated by subtracting the baseline (mock cell absorbance) from the values obtained from the infected wells. This subtraction ensured that any observed signal in the experiment wells was attributed to specific antigen–antibody interactions, thus enhancing the reliability and specificity of the assay. By using mock cells as background controls, we confirmed that the signals measured in the infected wells accurately reflected the presence of antibodies binding to the AHS virus antigens, rather than any non-specific interactions.

Table 1 and Table 2 present the OD readings from the checkerboard titration of the in-house indirect ELISA, where the coated antigen was derived from the monovalent live attenuated AHS vaccine. Antigen dilutions ranged from 1:20 to 1:160, and serum dilutions from 1:100 to 1:3200. The optimal OD was defined as twice the baseline, corresponding to antigen dilutions between 1:40 and 1:80, and serum dilutions between 1:800 and 1:1600. For this study, both monovalent and polyvalent antigens were diluted to 1:50, and serum samples were diluted to 1:1000 to evaluate their performance in comparison with the blocking ELISA.

The results of the in-house indirect ELISA used to detect antibodies in 94 seronegative and 94 seropositive horse serum samples were compared to those obtained using the blocking ELISA. The assay utilized both monovalent and polyvalent live attenuated AHS vaccines. Table 3 summarizes these comparisons, highlighting differences in antibody detection between the two assays. Additionally, sensitivity and specificity were evaluated for both vaccine types. The in-house ELISA demonstrated sensitivity values of 88.30% for monovalent vaccine and 87.23% for polyvalent vaccines, with specificity values of 67.02% and 84.04%, respectively, when compared to the blocking ELISA (Table 4).

To assess the specificity of the in-house ELISA and evaluate potential cross-reactivity, serum samples were tested for antibodies against equine encephalosis, equine influenza, and equine infectious anemia. All samples tested negative for these conditions, thereby confirming the assay’s specificity in detecting antibodies specific to African horse sickness.

## 4. Discussion

The routine measures for controlling and preventing AHS in epizootic countries include movement restrictions, quarantine, vector elimination, and vaccination, all aimed at limiting the spread of the disease [28]. In addition to these preventive measures, laboratory diagnosis is essential for demonstrating freedom from AHS infection in equine populations. It also plays a key role in evaluating eradication efforts, confirming clinical cases, estimating AHS prevalence, and assessing post-vaccination immunity [3,29]. This study aimed to compare the performance of monovalent and polyvalent antigens in an indirect ELISA. The results indicated that the analytical sensitivity of polyvalent antigen (87.23%) was not significantly different from that of the monovalent antigen (88.30%). However, the specificity of the polyvalent (84.04%) was significantly higher than that of the monovalent antigen (67.02%). The lower specificity of the monovalent antigen was likely due to an increased number of false positives. These findings suggest that impurities in live attenuated vaccines may contribute to non-specific immune responses or cross-reactivity, leading to a higher rate of false positives. The polyvalent antigen demonstrated higher analytical specificity, while the sensitivity of the monovalent antigen was not significantly different from that of the polyvalent antigen. This study supports the conclusion that the in-house indirect ELISA based on the polyvalent antigen offers a more specific and accurate approach to AHS antibody detection. These findings differ from previous studies, which showed that dot blotting with the monovalent antigen achieved greater specificity than the polyvalent antigen from the live attenuated AHS vaccine [25].

Serological surveillance of equine populations is essential for confirming the absence of AHSV transmission within a specific country or zone. The species selected for surveillance should reflect the local epidemiology of AHSV infection and the availability of equine populations. Management factors, such as insecticide use and animal housing, which may influence the likelihood of infection, should be considered when selecting equids for a surveillance system. Samples should be tested for AHSV antibodies, with positive results potentially arising from natural infection, AHSV vaccination, maternal antibodies, or issues related to test specificity [22]. While sera collected for other purposes may be utilized, the design of the survey must adhere to the principles required for statistically valid AHSV infection surveillance. Results from both random and targeted surveys provide reliable evidence of AHSV absence within a country or zone. Comprehensive documentation of the survey is necessary for the interpretation of results, which should be considered in the context of the movement history of the sampled animals. In AHSV-free zones, surveillance should focus on areas with the highest risk of transmission, based on prior surveillance data and other factors, including the zone boundaries. Depending on the epidemiology of AHSV, either random or targeted sampling is appropriate for selecting herds or individual animals for testing. Serological surveillance should be conducted in AHSV-free regions or zones situated at an adequate distance from infected areas, considering geographic, climatic, and infection history factors [28]. During the AHSV outbreak in Thailand, serotype 1 was confirmed [11,12,13]. In this study, serotype 1 was used for both monovalent and polyvalent vaccines during development and evaluation.

In this study, the performance of the in-house indirect ELISA was evaluated for its ability to detect antibodies against AHS, demonstrating satisfactory sensitivity and specificity comparable to that of the commercial blocking ELISA. To assess the precision of the assays, both intra-assay and inter-assay variations were analyzed (Table 5). The results for both monovalent and polyvalent antigens showed low intra-assay variability, indicating that the assays were reproducible under identical conditions. Intra-assay variability was assessed by testing duplicate samples on the same plate, and the low CV reflected high consistency within a single assay. Similarly, inter-assay variability was minimal, as evidenced by the consistent results obtained when the assays were repeated on different days. The reproducibility of the in-house indirect ELISA across multiple experimental runs demonstrates its reliability as a diagnostic tool. The minimal variability observed in both assays underscores the reliability of the in-house indirect ELISA in accurately detecting AHS antibodies. The low inter- and intra-assay CV values suggest that the assay can be confidently used for routine serodiagnosis testing, ensuring consistent results across multiple experiments and testing conditions.

In this study, negative sera were collected prior to the AHS outbreak in Thailand, while positive sera were obtained from AHS-vaccinated horses. All collected sera were validated as either negative or positive controls using a standard serological test, the blocking ELISA. This method is widely recognized as the standard for detecting AHS antibodies, as demonstrated by research groups such as Durán-Ferrer et al. [29] and the OIE [23]. Acknowledging the significance of accurate diagnosis, this study developed and tested an in-house ELISA using antigens derived from monovalent and polyvalent live attenuated AHS vaccines, comparing its performance to that of blocking ELISA. However, this study did not include sera from infected horses. Future research should incorporate samples from both naturally and experimentally infected horses to enhance diagnostic accuracy. Furthermore, purified AHSV antigens, such as whole virus, recombinant virus, or subunit virus antigens, should be utilized in future studies to confirm specificity and support the development of a commercial indirect ELISA.

## 5. Conclusions

The current study demonstrated that the in-house indirect ELISA is a valuable tool for detecting antibodies against AHS, providing a practical alternative for serodiagnosis. However, due to the potential for both false-positive and false-negative results, careful interpretation is required, particularly when assessing positive results in unvaccinated horses. The polyvalent antigen-based in-house indirect ELISA is a reliable alternative for AHS serodiagnosis, especially for vaccinated horses, where it offers improved specificity. Furthermore, this method is simpler, more cost-effective, faster, and more convenient than blocking ELISA and serum neutralization tests, making it a more practical choice for routine AHS surveillance.

## Figures and Tables

**Table 1:** Representative OD450 readings obtained for the checkerboard titration of the in-house indirect enzyme-linked immunosorbent assay coated antigen from the monovalent live attenuated African horse sickness vaccine, with dilutions ranging from 1:20 to 1:160 and negative horse serum dilutions ranging from 1:100 to 1:3200.

Serum		1:100		1:200		1:400		1:800		1:1600		1:3200	
Antigen		1	2	3	4	5	6	7	8	9	10	11	12
1:20	A	0.76	0.79	0.57	0.36	0.36	0.36	0.25	0.26	0.16	0.17	0.15	0.15
	B	0.83	0.83	0.62	0.65	0.42	0.40	0.30	0.30	0.20	0.22	0.17	0.18
1:40	C	0.73	0.74	0.55	0.56	0.40	0.38	0.28	0.28	0.18	0.17	0.14	0.16
	D	0.71	0.73	0.56	0.56	0.38	0.36	0.28	0.29	0.18	0.21	0.15	0.16
1:80	E	0.68	0.70	0.55	0.53	0.37	0.37	0.27	0.27	0.18	0.18	0.13	0.15
	F	0.68	0.70	0.53	0.53	0.37	0.40	0.28	0.32	0.20	0.26	0.17	0.18
1:160	G	0.66	0.70	0.54	0.55	0.36	0.37	0.26	0.25	0.15	0.16	0.10	0.12
	H	0.67	0.72	0.56	0.56	0.40	0.42	0.31	0.35	0.22	0.28	0.21	0.21

**Table 2:** Representative OD450 readings obtained for the checkerboard titration of the in-house indirect enzyme-linked immunosorbent assay coated antigen from the monovalent live attenuated African horse sickness vaccine, with dilutions ranging from 1:20 to 1:160 and positive horse serum dilutions ranging from 1:100 to 1:3200.

Serum		1:100		1:200		1:400		1:800		1:1600		1:3200	
Antigen		1	2	3	4	5	6	7	8	9	10	11	12
1:20	A	1.48	1.46	1.20	1.19	0.90	0.90	0.65	0.62	0.38	0.34	0.23	0.23
	B	1.42	1.49	1.24	1.25	0.96	1.02	0.68	0.68	0.42	0.40	0.26	0.29
1:40	C	1.35	1.35	1.10	1.09	0.82	0.80	0.55	0.55	0.37	0.37	0.24	0.24
	D	1.30	1.28	1.05	1.03	0.81	0.81	0.56	0.56	0.36	0.37	0.23	0.24
1:80	E	1.20	1.09	0.91	0.88	0.66	0.65	0.67	0.46	0.30	0.29	0.20	0.22
	F	1.21	1.07	0.88	0.90	0.65	0.66	0.49	0.56	0.34	0.35	0.23	0.22
1:160	G	0.97	0.87	0.90	0.70	0.50	0.49	0.35	0.34	0.22	0.21	0.14	0.13
	H	0.99	0.96	0.77	0.74	0.55	0.56	0.42	0.50	0.32	0.33	0.22	0.23

**Table 3:** The summary results of the in-house indirect enzyme-linked immunosorbent assay (indirect ELISA) using both monovalent and polyvalent live attenuated African horse sickness compared with the blocking enzyme-linked immunosorbent assay (bELISA) for antibody detection.

		Indirect ELISA (Monovalent)		Indirect ELISA (Polyvalent)	
		Positive	Negative	Positive	Negative
bELISA	Positive	83	11	82	12
	Negative	31	63	15	79

**Table 4:** The analytic sensitivity and specificity of the in-house indirect enzyme-linked immunosorbent assay (indirect ELISA) using both monovalent and polyvalent live attenuated African horse sickness compared with the blocking enzyme-linked immunosorbent assay (bELISA) for antibody detection.

	Indirect ELISA Monovalent	Indirect ELISA Polyvalent
Sensitivity (%)	88.30	87.23
Specificity (%)	67.02	84.04

**Table 5:** Intra- and inter-assay variability (CV) of the in-house indirect enzyme-linked immunosorbent assay (indirect ELISA) using both monovalent and polyvalent live attenuated African horse sickness.

Assay Type	Replicates Tested	CV (%)	Notes
Intra-assay (Monovalent antigen)	Duplicate samples within the same plate	5.2%	Consistency within a single experiment
Intra-assay (Polyvalent antigen)	Duplicate samples within the same plate	4.8%	Consistency within a single experiment
Inter-assay (Monovalent antigen)	Same samples, different days	6.3%	Reproducibility across multiple experiments
Inter-assay (Polyvalent antigen)	Same samples, different days	5.9%	Reproducibility across multiple experiments

## Data Availability

The data presented in this study are available free of charge for any user on request from the corresponding authors.
